# Immunotherapy as rescue for cardiomyopathy in Omenn syndrome

**DOI:** 10.70962/jhi.20250046

**Published:** 2025-06-23

**Authors:** Simon Boutinaud, Vincent Barlogis, Lionel Berthomieu, Montserrat Sierra Colomina, Marlène Pasquet

**Affiliations:** 1 Department of Pediatric Immunology, CHU Toulouse, Toulouse, France; 2 Department of Pediatric Immunology, Assistance publique - Hôpitaux de Marseille, Marseille, France; 3 Department of Pediatric Critical Care, CHU Toulouse, Toulouse, France; 4 Cancer Research Centre of Toulouse, UMR1037 Inserm, Toulouse, France

## Abstract

A 6-month-old infant with Omenn syndrome and severe hypertrophic cardiomyopathy showed significant clinical improvement following multimodal immunosuppression with anti–IL-5 and alemtuzumab. This targeted approach successfully stabilized the patient, providing a critical bridge to curative hematopoietic stem cell transplantation.

## Teaser

Omenn syndrome is a severe autosomal recessive combined immunodeficiency whose only curative treatment is allogeneic stem cell transplantation. We describe a rare case of infiltrative cardiomyopathy secondary to Omenn syndrome successfully treated by a combination of anti-IL5 antibodies and alemtuzumab, allowing successful HSCT.

Omenn syndrome (OS) is a severe combined immunodeficiency resulting from a restricted T cell repertoire characterized by skin rash, hepato-splenomegaly, and eosinophilia. Our understanding of this disease has not ceased to expand, thanks to the discovery of causal mutations in RAG-1 and RAG-2 genes, which are the most common causes of OS. They encode essential proteins in V(D)J recombination, essential to generate a wide variety of B and T cell receptors. In OS, recombination is impaired but not abolished, leading to B and T cell lymphopenia, with persisting T cell clones, which might survive and expand in the periphery and in the thymus (1). These T cell clones exhibit a strong restricted TCR heterogeneity and produce T-helper type 2 lymphocyte (Th2) cytokines, including IL-5, leading to hypereosinophilia, high serum level of IgE, and the possibility of lymphocytic and/or eosinophil infiltration of various organs, such as skin, liver, gut, and spleen, causing a graft-versus-host disease–like syndrome (1). Hematopoietic stem cell transplantation (HSCT) remains the only curative therapeutic option for patients with OS, and few articles focus on other therapies to manage life-threatening complications before HSCT.

**Figure 1.**
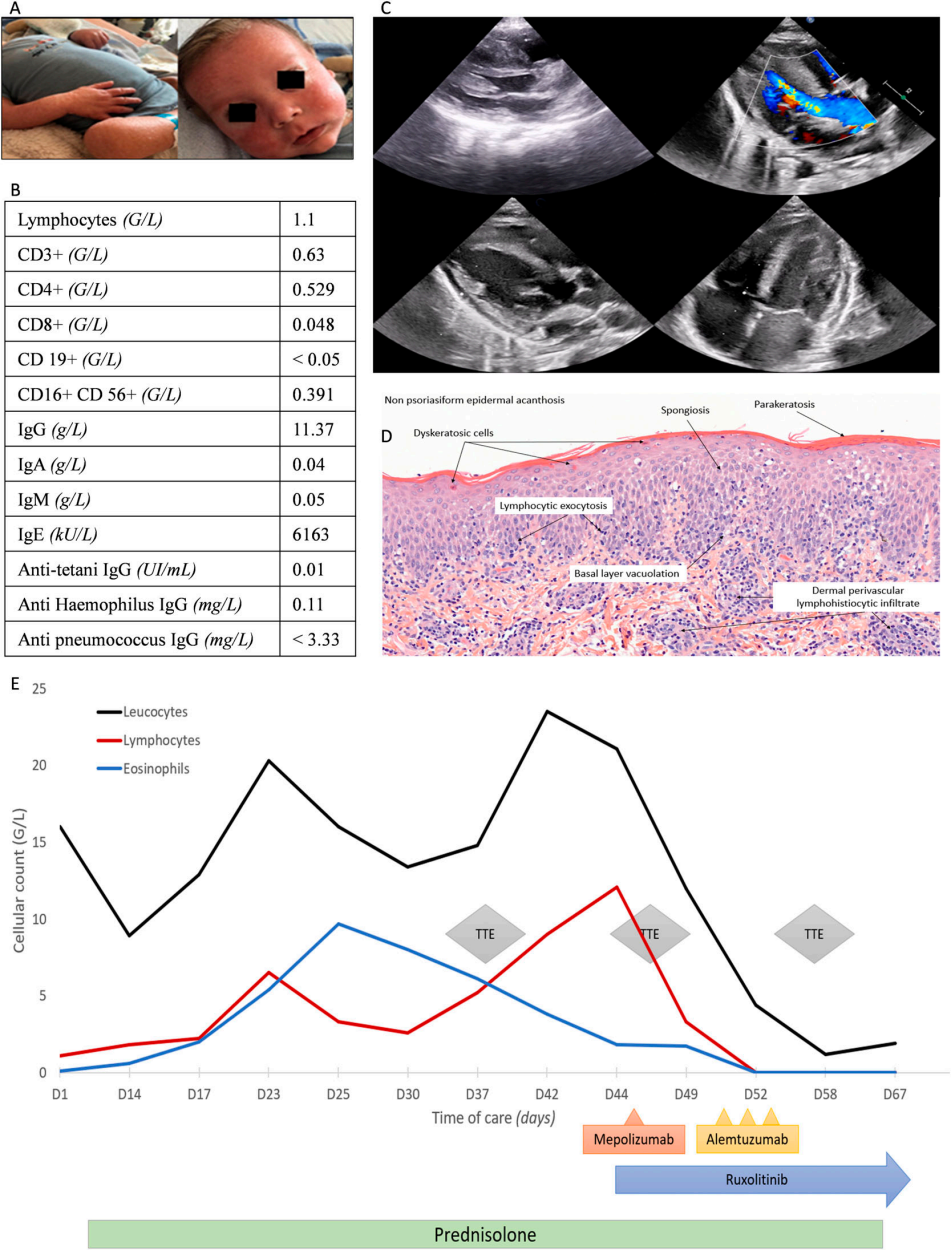
**Identification of skin and cardiac involvement in a child with OS. (A)** Diffuse erythroderma of the limbs and face at diagnosis. **(B)** Immunological characteristics of patient at baseline diagnosis. **(C)** TTE before transplantation (upper panel) and after transplantation (lower panel). Left upper part showed interventricular septum thickening in a short axis, and right upper part intraventricular acceleration in a long axis. On the left lower part, long axis showed no ventricular hypertrophy after transplantation, with normal myocardial diameters in a four-cavity view at the right lower part. **(D)** Skin biopsy showing a superficial derm and perivascular infiltrate consisting of histiocytes and a mixture of CD4 and CD8 lymphocytes. **(E)** Schematic representation of the patient’s clinical history.

We report the case of a 6-mo-old infant, who developed a severe hypertrophic cardiomyopathy related to OS, which improved after multimodal immunosuppression, allowing a bridge to HSCT. This child was born from consanguineous parents and presented with *Pneumocystis* pneumonia at 6 mo, requiring hospitalization in pediatric intensive care unit (PICU) for respiratory support. Medical history showed daily diarrhea and a failure to thrive since the age of 2 mo. Clinical examination revealed diffuse maculopapular rash with xerosis (Fig. 1 A), hepatomegaly, and axillary and inguinal lymphadenopathies. Echocardiography at day 1 (referring to the day of admission in the PICU) was normal. Immunophenotyping revealed CD4 and CD8 T cell lymphopenia without response after mitogen stimulation, profound B cell lymphopenia, and normal natural killer (NK) count. Immunoglobulins were absent for IgA and IgM and elevated for IgG. The child was breastfed. Vaccinal responses to tetanus, *Haemophilus*, and pneumococcus were negative (Fig. 1 B). Of note, IgE were elevated with an increased eosinophilia above 2 g/liter (normal range: 0–0.5 g/liter) at day 17 (Fig. 1 B). Nasal swab was positive for respiratory syncytial virus and parainfluenzae I, and stools were positive for astrovirus. Skin biopsy revealed a CD4/CD8 lymphocytic infiltrate with no B lymphocytes, associated with intraepidermal exocytosis, keratinocytic necrosis, and discrete epidermal acanthosis (Fig. 1 D) without eosinophilic infiltration. OS was highly suspected and genetically confirmed after next-generation sequencing of the index case (inborn error of immunity panel), revealing a homozygous mutation in the exon 2 of the RAG2 gene (NM_000536.3:c.1090_1093del, NM_000536.3:p.(Asn364Valfs*79)) present in both of the child’s parents in a heterozygous state. Prednisolone, set up at 2 mg/kg/day from day 1 because of oxygen requirement, was progressively diminished.

HSCT was planned, but pretransplant transthoracic echocardiogram (TTE) on day 37 revealed severe biventricular hypertrophy with intra-left ventricular obstruction (Fig. 1 C right upper part and Fig. 1 C left upper part), along with sinus tachycardia on the electrocardiogram. Magnetic resonance imaging (MRI) was performed and was suggestive of infiltrative cardiomyopathy. After ruling out differential diagnoses, lymphocytic and/or eosinophilic infiltrative cardiomyopathy remained the main hypothesis. Notably, a whole-exome sequencing in trio with parents was performed to exclude other etiologies of congenital hypertrophic cardiomyopathy. The patient was started on beta-blocker treatment (propranolol up to 5 mg/kg/day), and prednisolone dose was increased back to 2 mg/kg/day with a worsening in tachycardia and myocardial hypertrophy. Eosinophil count kept rising up to 9.7 g/liter despite corticosteroid treatment. We introduced ruxolitinib on day 44 as steroid sparing (5 mg every 12 h, normal residual range), followed by one dose of mepolizumab (20 mg) on day 46 and daily alemtuzumab infusion from day 51 to 53 (0.5 mg/kg on day 51, 1 mg/kg on days 52–53). First and second doses of alemtuzumab resulted in grade II anaphylactic reactions with favorable outcome. Blood count showed resolution of hypereosinophilia and complete lymphocytic depletion on day 52. Repeated TTE on day 56 revealed disappearance of ventricular obstruction and mild improvement of biventricular hypertrophy. Lymphopenia persisted over time, and eosinophil count remained null. Erythroderma and lymphadenopathies disappeared, allowing discontinuation of prednisolone on day 67 and ruxolitinib on day 88 (Fig. 1 E). Hepatomegaly persisted until HLA-matched unrelated donor HSCT took place on day 108 from marrow as donor source. The child received a reduced intensity regimen: four doses of 40 mg/m^2^ of fludarabine; four doses of 1.2 mg/kg of busulfan; three doses of 2.5 mg/kg of antilymphocyte globulin. Graft-versus-host disease prophylaxis consisted in ciclosporin and methotrexate. The clinical course of our patient was favorable during the procedure, with no signs of heart failure before, during, or after HSCT. Of note, hyperhydration was well tolerated.

Chimerism on whole blood was full donor at 3 mo. It remained full donor on CD3^+^ cells despite falling to 42% on whole-blood cells at 12 mo. Immunological and hematological reconstitution were correct with a normal proportion of T, B, and NK cells 1 year after HSCT with 48% naïve CD4^+^ T cells and 16% of naïve CD8^+^ T cells. Control TTE 3, 6, and 12 mo after HSCT were normal (Fig. 1 C left lower part and Fig. 1 C right lower part). With a follow-up of 13 mo after HSCT, the child is doing well and asymptomatic.

OS is a severe autosomal recessive combined immunodeficiency whose only curative treatment is HSCT. RAG-1 or RAG-2 mutations cause a defect in V(D)J recombination but do not totally prevent it, generating an oligo-clonal T lymphocyte repertoire infiltrating skin, liver, or digestive tract. This predominantly Th2 expansion is responsible for IL-5–mediated hypereosinophilia, which usually leads to organ infiltration, such as skin, digestive tract, or liver. We described here a very rare case of infiltrative cardiomyopathy secondary to OS, which was successfully treated by a combination of anti-IL5 antibodies and alemtuzumab, allowing successful HSCT.

To our knowledge, only one case of OS with myocardial hypertrophy has been described in the literature. It involved a 5-mo-old infant with biventricular myocardial hypertrophy without histopathological diagnosis. The child underwent HSCT without cardiomyopathy-specific pretransplant treatment. He survived but developed severe pulmonary veno-occlusive disease immediately after transplant, leading to several episodes of cardiogenic shock (2). 30 years later, we tried to set up a specific treatment based on the disease’s pathogenesis in order to safely transplant the infant. Cardiac MRI was suggestive of an infiltrative process but could not assess the eosinophilic or lymphocytic origin of the cardiomyopathy. Trans-jugular biopsy could have provided its definitive etiology, but the procedure was too risky.

First of all, we started treatment with mepolizumab, a monoclonal antibody that targets IL-5. It allows quick eosinophil count decrease in hypereosinophilic syndromes and can be used in second-line therapy for eosinophilic cardiopathy (3). Hypereosinophilic cardiac involvement leads to acute myocarditis, intracardiac thrombosis, and progresses to myocardial fibrosis. None of these features were found in our patient. Given that the eosinophil-mediated nature of this myocardial hypertrophy remained controversial and that cardiomyopathy was life-threatening, we introduced a second monoclonal antibody directed against lymphocytes (as well as eosinophils) without waiting to evaluate long-term response to mepolizumab. Alemtuzumab targets CD-52, a surface glycoprotein expressed on lymphocytes, macrophages, monocytes, and eosinophils. It has been successfully used in multiple sclerosis, solid organ transplant, and hemophagocytic lymphohistiocytosis, allowing quick lymphocytic depletion. It has also been used in corticosteroid-refractory hypereosinophilic syndromes in adults (4) by providing direct toxicity on eosinophilic lineage and impairing IL-5 production by T cell destruction. The tolerance was good with no cytokine release syndrome, no viral complications, and complete lymphocyte depletion achieved after first dose. Clinical symptoms (tachycardia) and TTE improved in the week following treatment allowing HSCT. Ruxolitinib is a JAK inhibitor already used in neoplasms with eosinophilia and tyrosine kinase gene fusion, but it has rarely been reported in treatment of other hypereosinophilic syndrome. Despite its potential effect on eosinophil and lymphocyte count, we believe its definitive contribution cannot be assessed as the duration of monotherapy was too short (5).

To conclude, we believe that combination of ruxolitinib, anti-IL5, and anti-CD52 therapy allowed myocardium hypertrophy’s regression, therefore confirming its infiltrative nature. According to clinical features and quick improvement after alemtuzumab, it is highly presumed that this hypertrophy was caused by lymphocytic rather than eosinophilic infiltration, even if we cannot definitively conclude on this point. We report the first case of a 6-mo-old child successfully treated by alemtuzumab and mepolizumab for infiltrative cardiomyopathy linked to OS, allowing definitive by HSCT without pulmonary or cardiac sequelae.
